# Supplementary material for: Changes in the prevalence of diabetes, prediabetes and associated risk factors in rural Baluchistan; a secondary analysis from repeated surveys (2002–2017)
Source: PLoS One. 2023 Apr 20;18(4):e0284441. doi: 10.1371/journal.pone.0284441 (PMC10118118; doi:10.1371/journal.pone.0284441)
Supplement: Attachment — Submitted filename: Response to Reviewer (1).docx [file pone.0284441.s001.docx]

**Response to Reviewer**

We all authors thank to consider and review our manuscript. We tried our best to incorporate valuable suggestions in our manuscript.

I commend that the topic of the paper is interesting, especially for the alarming increase in a rural population. However, my own reading raised a number of concerns which need to be addressed before any final decision is made.

First, the Methods section needs clarification. For instance, it is not clear which outcomes were collected in three surveys and which ones were not.

Response: Changes suggested are made in the manuscript and highlighted.

Instances are shown in Figure 1E and 1F where only two surveys are presented.

**Response:** Given the variables of central obesity and dyslipidemia were not obtained in survey year 2001-02 through the suggested changes suggested are being made in the methodology and highlighted.

Second, authors refer to "pooled prevalence", it seems that they just collapsed pre and diabetes categories?

**Response:** Yes, it gives collapsed prevalence of pre and diabetes category. However, we decided to keep the figures 2A and 2B , to understand the prevalence by gender and age. The term pooled is removed from the figure as it is not suited. Changes suggested are being made in the manuscript and highlighted.

Third, the methods section is fuzzy the way it stands now. It is unclear which are the outcomes and associated factors in the paper. This section needs to reworked to reflect the standard of PLOS One. Third, this section is unclear regarding the sampling design in the three surveys, and how the sampling design was taken into account the estimations. Without this information or clarification, it is not possible for the readers to trust the estimates.

**Response:** Changes suggested are being made in the manuscript and highlighted.

Fourth, I wonder how readers could trust estimates from the 2001-2002 survey given tremendous differences with 2009-2010 and 2016-2017 surveys. The estimates from these surveys cannot be trusted without further explanations of these differences. What happened between 2001-2002 survey and subsequent surveys. I question what has changed in the Pakistani rural context to see such a shift in only 10 years. Usually, such changes are expected on long-run but short- or mid-run. As a consequence, most differences in percentage points between 2001-2002 and 2016-2017 are above 10 points and combined obesity, over 15 years, increased by 72 percentage points. This is impressive and questionable at same time (Table 2). Surprisingly, the Discussion section is silent about convincing explanation of this health catastrophe. This is where authors need to be more creative and build from their knowledge of the ground in the Pakistani context to explain to the readers what happened to explain these findings.

**Response:** Changes suggested to improve the discussion are being made in the manuscript and highlighted.

Finally, my fears are consolidated by Table 3 where only a few variables showed statistical significance with the outcomes of the paper. Why?

**Response:**

**Though the odds prediabetes and diabetes had weak significance with covariates, but the cardiometabolic risk factors were strongly associated with hypertension, central obesity and dyslipidemia which implies policy decisions to tackle modifiable risk factors.**

Please submit your revised manuscript by February 22, 2023. If you will need more time than this to complete your revisions, please reply to this message or contact the journal office at [plosone@plos.org](mailto:plosone@plos.org). When you're ready to submit your revision, log on to <https://www.editorialmanager.com/pone/> and select the 'Submissions Needing Revision' folder to locate your manuscript file.

Please include the following items when submitting your revised manuscript:

- A rebuttal letter that responds to each point raised by the academic editor and reviewer(s). You should upload this letter as a separate file labeled 'Response to Reviewers'.
- A marked-up copy of your manuscript that highlights changes made to the original version. You should upload this as a separate file labeled 'Revised Manuscript with Track Changes'.
- An unmarked version of your revised paper without tracked changes. You should upload this as a separate file labeled 'Manuscript'.

If you would like to make changes to your financial disclosure, please include your updated statement in your cover letter. Guidelines for resubmitting your figure files are available below the reviewer comments at the end of this letter.

If applicable, we recommend that you deposit your laboratory protocols in [protocols.io](http://protocols.io/) to enhance the reproducibility of your results. Protocols.io assigns your protocol its own identifier (DOI) so that it can be cited independently in the future. For instructions see: <https://journals.plos.org/plosone/s/submission-guidelines#loc-laboratory-protocols>. Additionally, PLOS ONE offers an option for publishing peer-reviewed Lab Protocol articles, which describe protocols hosted on [protocols.io](http://protocols.io/). Read more information on sharing protocols at <https://plos.org/protocols?utm_medium=editorial-email&utm_source=authorletters&utm_campaign=protocols>.

We look forward to receiving your revised manuscript.

Kind regards,

Zacharie Tsala Dimbuene, Ph.D.
Academic Editor
PLOS ONE

Journal requirements:

When submitting your revision, we need you to address these additional requirements.

1. Please ensure that your manuscript meets PLOS ONE's style requirements, including those for file naming. The PLOS ONE style templates can be found at

<https://journals.plos.org/plosone/s/file?id=wjVg/PLOSOne_formatting_sample_main_body.pdf> and

<https://journals.plos.org/plosone/s/file?id=ba62/PLOSOne_formatting_sample_title_authors_affiliations.pdf>

2. PLOS requires an ORCID iD for the corresponding author in Editorial Manager on papers submitted after December 6th, 2016. Please ensure that you have an ORCID iD and that it is validated in Editorial Manager. To do this, go to ‘Update my Information’ (in the upper left-hand corner of the main menu), and click on the Fetch/Validate link next to the ORCID field. This will take you to the ORCID site and allow you to create a new iD or authenticate a pre-existing iD in Editorial Manager. Please see the following video for instructions on linking an ORCID iD to your Editorial Manager account: <https://www.youtube.com/watch?v=_xcclfuvtxQ>.

3. Please ensure that you refer to Figure1 & 2 in your text as, if accepted, production will need this reference to link the reader to the figure.

Reviewers' comments:

Reviewer's Responses to Questions

**Comments to the Author**

1. Is the manuscript technically sound, and do the data support the conclusions?

The manuscript must describe a technically sound piece of scientific research with data that supports the conclusions. Experiments must have been conducted rigorously, with appropriate controls, replication, and sample sizes. The conclusions must be drawn appropriately based on the data presented.

Reviewer #1: Yes

Reviewer #2: Yes

2. Has the statistical analysis been performed appropriately and rigorously?

Reviewer #1: Yes

Reviewer #2: Yes

3. Have the authors made all data underlying the findings in their manuscript fully available?

The [PLOS Data policy](http://www.plosone.org/static/policies.action#sharing) requires authors to make all data underlying the findings described in their manuscript fully available without restriction, with rare exception (please refer to the Data Availability Statement in the manuscript PDF file). The data should be provided as part of the manuscript or its supporting information, or deposited to a public repository. For example, in addition to summary statistics, the data points behind means, medians and variance measures should be available. If there are restrictions on publicly sharing data—e.g. participant privacy or use of data from a third party—those must be specified.

Reviewer #1: Yes

Reviewer #2: Yes

4. Is the manuscript presented in an intelligible fashion and written in standard English?

PLOS ONE does not copyedit accepted manuscripts, so the language in submitted articles must be clear, correct, and unambiguous. Any typographical or grammatical errors should be corrected at revision, so please note any specific errors here.

Reviewer #1: Yes

Reviewer #2: Yes

5. Review Comments to the Author

Please use the space provided to explain your answers to the questions above. You may also include additional comments for the author, including concerns about dual publication, research ethics, or publication ethics. (Please upload your review as an attachment if it exceeds 20,000 characters)

Reviewer #1: Diabetes is one of the biggest challenges for the public health and the number of people affected by this disease still increases. There is still a big gap in diabetes management between high and low-income countries. It has been reported that approximately 80% of adults with diabetes worldwide live in low-income and middle-income countries.
Therefore, despite the fact that presented study comes from one region with small study group, I think it is valuable to present. Most pre-existing studies come from urban populations or high income countries, so in that way presented study adds to the existing evidence and helps to draw attention to the problem of diabetes in rural areas. In my opinion the article is well-written. I have some minor comments:
I think it would be helpful if overall number of participants was mentioned in the abstract and the methods section.

**Response:** Changes suggested are made in the manuscript and highlighted.

It is stated that “BMI <23 kg/m2 was considered as normal, ≥23-24.9 kg/m2 as overweight and ≥25 kg/m2 as obese”. The citation [19] at the end of the sentence doesn’t lead to the source of the criteria and they differ from the widely recognizable WHO norms. The reference should indicate the guidelines for BMI, if the Authors chose the norms for Asia-Pacific region, it should be clearly explained.

**Response:** Changes suggested are made in the manuscript and highlighted.

The abstract should be improved, as there are some nominal sentences.

**Response:** Changes suggested are made in the manuscript and highlighted.

Reviewer #2: In page 3, line 65, It is mentioned that population of Baluchistan (85% rural, 38% urban) which needs correction

**Response:** Changes suggested are made in the manuscript and highlighted.

In discussion, page 18, findings of tobacco consumption at national level like in NCDs survey [<https://pubmed.ncbi.nlm.nih.gov/29658619/>] and Global Adult Tobacco Survey [<https://pubmed.ncbi.nlm.nih.gov/29059338/>] may be discussed in detail that why it is different from general population.

**Response:** Changes suggested are made in the manuscript and highlighted.

6. PLOS authors have the option to publish the peer review history of their article ([what does this mean?](https://journals.plos.org/plosone/s/editorial-and-peer-review-process#loc-peer-review-history)). If published, this will include your full peer review and any attached files.


If you choose “no”, your identity will remain anonymous but your review may still be made public.


**Do you want your identity to be public for this peer review?** For information about this choice, including consent withdrawal, please see our [Privacy Policy](https://www.plos.org/privacy-policy).

Reviewer #1: No

Reviewer #2: **Yes:** Dr. Ibrar Rafique
